# EV duty vehicles: Features and functions of ciliary extracellular vesicles

**DOI:** 10.3389/fgene.2022.916233

**Published:** 2022-08-19

**Authors:** Ludovic Vinay, Clémence Belleannée

**Affiliations:** Faculty of Medicine, Université Laval, CHU de Québec Research Center, Québec, QC, Canada

**Keywords:** ciliary extracellular vesicles, primary cilia, intercellular communication, ectosomes, motile cilia, markers, ectocytosis

## Abstract

The primary cilium is a microtubule-based organelle that extends from a basal body at the surface of most cells. This antenna is an efficient sensor of the cell micro-environment and is instrumental to the proper development and homeostatic control of organs. Recent compelling studies indicate that, in addition to its role as a sensor, the primary cilium also emits signals through the release of bioactive extracellular vesicles (EVs). While some primary-cilium derived EVs are released through an actin-dependent ectocytosis and are called ectosomes (or large EVs, 350–500 nm), others originate from the exocytosis of multivesicular bodies and are smaller (small EVs, 50–100 nm). Ciliary EVs carry unique signaling factors, including protein markers and microRNAs (miRNAs), and participate in intercellular communication in different organism models. This review discusses the mechanism of release, the molecular features, and functions of EVs deriving from cilia, based on the existing literature.

## Introduction

Extracellular vesicles (EVs) are small elements released from the cell into the extracellular space and delimited by a lipid bilayer (Théry et al., 2018). Previously considered artefactual cellular debris (Cocucci et al., 2009), these particles are now recognized as potent means of intercellular communication through the transport of bioactive molecules, including proteins, lipids and non-coding RNA, from a donor to a recipient cell. Since the release of EVs and their molecular cargo is tightly associated with the patho-physiological status of the donor cells and tissues, there is an increasing interest in using EVs from bodily fluids in the non-invasive diagnosis of diseases. While the number of studies reporting on EVs has increased exponentially, the described diversity of these cellular particles has also expanded, rendering their classification difficult. According to the nomenclature established by the International Society of Extracellular Vesicles (ISEV), EVs are separated into two main categories: large EVs and small EVs. **Large EVs** (sometimes referred to as ectosomes, microvesicles or microparticles) are extracellular vesicles of 100–500 nm, generated by budding from the plasma membrane and released into the cell environment, while **small EVs** (also referred to as exosomes depending on their molecular markers) measure between 50 and 150 nm and are initially formed inside multivesicular bodies (MVB) before being released outside the cell by fusion of the MVB with the plasma membrane (Meldolesi, 2018). It is worth mentioning that this nomenclature is based chiefly on research on EVs deriving from the cellular cytoplasm, from human and mouse models. Over the past 10 years, a new class of EVs has come under the spotlight of leading laboratories that specialize in ciliary organelles: the ciliary EVs (Wood and Rosenbaum, 2015; Carter and Blacque, 2019; Nachury and Mick, 2019). While it is unclear what category ciliary EVs belong to, small and/or large ectosome-like EVs protruding from the base, the axonemal membrane, or the tip of cilia have been observed in multiple species (Table 1). The most compelling findings of ciliary EVs come from the studies of model organisms such as *Chlamydomonas* and *Caenorhabditis elegans* (for review, see Wood et al., 2013; Wang and Barr, 2018), where ciliary EVs participate in the control of mating behavior (Wang et al., 2014; Maguire et al., 2015; Garcia et al., 2018; Carter and Blacque, 2019). The present article provides an overview of the seminal knowledge on ciliary EVs that comes from organism models and puts emphasis on recent studies that delineate the features and mode of release of ciliary EVs from mammalian ciliated cells. Functional investigations into the contribution of ciliary EVs to intercellular communication and the control of biological systems in mammals are still lacking. However, we extend the current knowledge of ciliary EVs to discuss their potential role and use in the context of human diseases, including cilia-related diseases called ciliopathies.

**TABLE 1:** Microscopic analysis revealed the presence of (A) cilia deformations (*e.g.,* bulb at the tip, membrane bud); (B) vesicles or vesicle-like structures associated with cilia (C) ciliary EVs generated at the ciliary tip (D) alongside the axoneme, or (E) the ciliary base.

*Source of ciliary EVs*		*Cilia*	*Models*	*EV markers*	*Imaging techniques*	*References*
***(A) Vesicular structures associated with cilia***	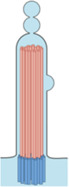	PC	Mouse neuroepithelial cells	Prominin-1	TEM	Dubreuil et al. (2007)
		PC	Inpp5e^Δ/Δ^ cells lining a mouse kidney cyst	-	TEM	Jacoby et al. (2009)
		PC	RPE-hTERT	Smo	FBM	Huang et al. (2016)
		PC	Prom1 K138Q mutation in MDCK cells	Prominin-1	SEM	Singer et al. (2019)
***(B) Vesicles associated with cilia***	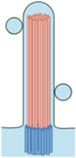	PC	Pkhd1^del2/del2^ mouse cholangiocyte	-	TEM	Woollard et al. (2007)
		PC	Pkhd1^del2/del2^ and WT mouse cholangiocyte	Polycystin 1	TEM	Hogan et al. (2009)
		PC	IMCD-3 and rat biliary epithelial cells	PKD proteins	SEM	
		PC	PCK rat cholongiocyte, WT and Pkhd1^del2/del2^ mouse cholangiocyte	-	SEM, TEM	Masyuk et al. (2010)
		PC	MEF	-	SEM	Pampliega et al. (2013)
		PC	MDCK	Smo, Sec10	FBM	Chacon-Heszele et al. (2014)
		MC	Turtle ductuli efferentes epithelial cells	-	TEM	Tarique et al. (2019)
		PC	MDCK-II	-	Cryo-ET	Kiesel et al. (2020)
***(C) Ciliary EV released at the cilia tip***	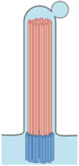	MC	*Chlamydomonas reinhardtii*	***-***	Cryo-EM, SEM	Bergman et al. (1975)
		MC	*Chlamydomonas reinhardtii, Chlamydomonas moewusii* strain cc957	VLE protease	TEM, DICM	Wood et al. (2013)
		PC	RPE1	CEP162	SEM, FBM	Wang et al. (2013)
		PC	*C. elegans* WT and mutants	LOV-1, PKD-2	TEM, ET	Wang et al. (2014)
				PKD-2	TEM, FBM	Maguire et al. (2015)
				PKD-2	TEM, ET, FBM	O’Hagan et al. (2017)
		MC	*Chlamydomonas reinhardtii*	-	TEM	Dentler, (2013)
						Cao et al. (2015)
		MC	*Chlamydomonas* WT and mutant strains	PDCD6	FBM	Long et al. (2016)
		PC	Arl6^−/−^ IMCD-3, β-arrestin2^−/−^ mIMCD-3	GPCRs	FBM	Nager et al. (2017)
		PC	NIH/3T3, mIMCD-3, hTERT RPE-1, Inpp5e^−/−^ and Inpp5e^+/-^ MEF	Arl13b, 5HT_6_	FBM	Phua et al. (2017)
		PC	Patient-derived glioblastoma cells	Arl13b	FBM	Hoang-Minh et al. (2018)
		PC	RPE1	Smo	FBM	Wang et al. (2019)
		PC	ICK-KO hTERT-RPE-1	Arl13b, IFT88	FBM	Nakamura et al. (2020)
		PC	*C. elegans* WT and mutants	PKD-2, CIL-7	TEM, ET, FBM	Akella et al. (2020)
				TSP-6, TSP-7	FBM	Razzauti and Laurent, (2021)
(***D***) ***Ciliary EV released alongside the axoneme***	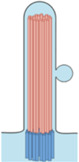	MC	*Trypanosoma brucei*	***-***	TEM, FBM, DICM	Szempruch et al. (2016b)
		PC	Rds^−/−^ mouse	-	TEM	Salinas et al. (2017)
		PC	Kidney cell lines	-	ET	Sun et al. (2019)
(***E***) ***Ciliary EV released at the ciliary base***	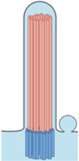	MC	*Chlamydomonas* WT and mutant strains	Flagellar membrane glycoproteins	FBM	Bloodgood et al. (1986)
		PC	*C. elegans* WT and mutants	LOV-1, PKD-2, CIL-7	TEM, ET	Wang et al., 2014, 2021a
				PKD-2, CIL-7	TEM, ET, FBM	Silva et al. (2017)
						Akella et al. (2020)
				TSP-6, TSP-7	FBM	Razzauti and Laurent, (2021)

PC, primary cilia; MC, motile cilia; WT, wild-type; PCK, rat model of polycystic kidney disease; mIMCD-3, mouse inner medullary collecting duct-3; MEF, mouse embryonic fibroblast; NIH/3T3, fibroblast cell line that was isolated from a mouse NIH/Swiss embryo; hTERT RPE-1, human telomerase reverse transcriptase immortalized retinal pigment epithelial cell; MDCK, Madin-Darby canine kidney cells; DICM, differential interference contrast microscopy; TEM, transmission electron microscopy; SEM, scanning electron microscope; FBM, fluorescence-based microscopy; ET, electron tomography; Cryo-EM, cryogenic electron microscopy; Cryo-ET, cryo-electron tomography.

## Cilia as a source of EVs

The cell plasma membrane is the interface between the cell body and the cell environment that controls many cellular processes, including cell volume, motility, adhesion, and quorum sensing. A large variety of membrane protrusions exist to 1) increase the surface of exchanges between the internal and external cellular environment (*e.g.,* microvilli, microplicae); 2) generate subcellular compartments enriched in receptors and signaling factors to sense the extracellular environment (*e.g.,* primary cilia, filopodia); or 3) control the fluid turbulence in the cell surrounding environment (*e.g.,* motile cilia). Beyond their classical role in cell sensing, accumulating evidence suggests that these membrane protrusions are cellular regions specialized in the release of EVs, which could mediate communication between cells (Rilla, 2021).

### Motile cilia and primary cilia

Cilia are microtubule-based organelles that emerge from the cell surface to sense and control the extracellular microenvironment. Cilia are composed of an axoneme that extends from a basal body. A transition zone forms a structural gate with Y-shaped connectors between the basal body and the axoneme to control protein entry into the cilium, thus forming a separate subcellular compartment (Garcia-Gonzalo and Reiter, 2012; Li et al., 2012; Takao and Verhey, 2016). From this common archetype, different types of cilia can be distinguished based on their ultrastructure. For instance, the axoneme of motile cilia consists of nine pairs of microtubules surrounding two central singlet microtubules (Fisch and Dupuis-Williams, 2011; Brooks and Wallingford, 2014; Spassky and Meunier, 2017). Along the axoneme, dynein ATPase motor proteins ensure microtubule sliding responsible for ciliary beating, and the intraflagellar transport system (IFT) participates in ciliogenesis (Jordan and Pigino, 2021). The IFT is a critical molecular device for the assembly and maintenance of the primary cilium, allowing bidirectional movement between the base and tip of structural and signaling components. Organized into repeating structures called IFT trains, IFT-A and IFT-B protein complexes act as adapters between the cargoes to be moved and the molecular motors, kinesin-2 (anterograde motor) and dynein-2 (retrograde motor), respectively. In addition to these two types of IFT trains, the BBsome is an octameric complex of Bardet–Biedl syndrome (BBS) proteins that acts as an adapter between the IFT complexes and the cargoes, especially G protein-coupled enriched receptors (GPCRs) (Jordan and Pigino, 2021). Unlike motile cilia, primary cilia lack dynein arms and central microtubules, giving this type of cilium primarily sensory attributes rather than a mechanical beating function (Singla and Reiter, 2006; Satir, 2017; Spasic and Jacobs, 2017). While non-motile primary cilia are solitary organelles that protrude from most post-mitotic cells in pluricellular organisms (Satir et al., 2010; Smith et al., 2020), motile cilia are tufted on the surface of epithelial cells and are found in limited body locations, including the brain, the respiratory and reproductive tracts (Spassky and Meunier, 2017). In these particular regions, the whip-like motion of motile cilia generates fluid flow over the apical surface of the epithelium and is responsible for major physiological functions, *e.g.,* flow of cerebrospinal fluid in the brain, clearing mucus from airways, transporting oocytes in the oviducts, and maintaining sperm in suspension in the efferent ductules to facilitate their concentration (Spassky and Meunier, 2017; Yuan et al., 2021;). Some motile cilia, such as the sperm flagellum or *Chlamydomonas* flagella, exist as solitary or paired organelles acting as a cell propellor ( Silflow and Lefebvre, 2001; Miki and Clapham, 2013 Bertiaux and Bastin, 2020 ). In contrast to the stirring properties of motile cilia, non-motile primary cilia are highly potent chemo- and mechano-sensors of the extracellular environment. For instance, primary cilia control cell proliferation, differentiation, and polarity in response to fluid shear stress and/or the presence of agonists of different signaling pathways (*e.g.,* Hedgehog, PDGF, Wnt) in the cell surrounding environment (Satir et al., 2010; Pala et al., 2017; Wheway et al., 2018; Anvarian et al., 2019).

### Ciliary EVs from motile and primary cilia

Both non-motile primary cilia and motile cilia are mostly located at the apical pole of the cells and form a privileged surface of exchange between the cell cytosol and the extracellular environment. Over the past 15 years, microscopic studies performed on model organisms and mammalian cells have revealed the presence of cilia deformations (*e.g.,* bulb at the tip, membrane bud) (Table 1A), vesicle-like elements associated with the cilium (Table 1B), which evoke the possible capacity of cilia to receive and/or emit ciliary EVs, or the presence of EVs released into the extracellular space (Table 1C-E). The detection of ciliary elements from different biological fluids has further supported the association between cilia and EVs (Marzesco et al., 2005; Dubreuil et al., 2007; Hogan et al., 2009). In addition to improving our comprehension of fundamental cellular biology, these findings open the possibility of applications for non-invasive diagnosis of ciliary dysfunctions and related diseases in humans (Mohieldin et al., 2021a). The release of EVs from motile cilia has mainly been studied in the unicellular green alga *Chlamydomonas* and the parasite *Trypanosoma brucei*. In the first organism, EVs are released from the tip and the base of the flagellum (references in Tables 1C,E), while in the second, they come from the nanotubes that bud from flagellar membrane (Szempruch et al., 2016b). To our knowledge, only one study reported the presence of EVs potentially deriving from motile cilia in vertebrates (Tarique et al., 2019). In this report on turtle efferent ductules, the authors used Transmission Electron Microscopy (TEM) to show the presence of EVs associated with motile ciliary axonemes and covered by the ciliary membrane. Additional studies are needed to validate this model and explore the possible role of these ciliary EVs in soma-sperm cell communication. In contrast, the role of primary cilia as a source of EVs in *Caenorhabditis elegans* ciliated sensory neurons and a large variety of ciliated cell lines has received greater study during the past years (for review, see Garcia et al., 2018; Carter and Blacque, 2019; Nachury and Mick, 2019). It appears that ciliary EVs can be generated from different sites of the cilium: at the ciliary tip (references in Table 1C), alongside the axoneme (references in Table 1D), or at the ciliary base, also called periciliary membrane compartment (PCMC) in *C. elegans* (references in Table 1E). While ciliary EVs and cytosolic EVs have different compositions and potentially different functions (Mohieldin et al., 2021b; Volz et al., 2021), very little is known about the differences between subpopulations of ciliary EVs from distinct regions of the primary cilia. The best-known example is in *C. elegans.* In this species, EVs released at the tips of primary cilia of sensory neurons are found in the surrounding extracellular environment, whereas EVs from the PCMC are released into the lumen surrounding the PCMC before being phagocytized by neighboring glial cells (Razzauti and Laurent, 2021). These two EVs subpopulations exert different functions (see below). In mammals, it remains to be determined whether these different origins are associated with different types of ciliary EVs with distinct compositions and functions, or differ according to the species or biological system studied.

## Functions of ciliary EVs

According to functional studies performed thus far on model organisms and mammalian cells, ciliary EVs may participate in intercellular communication between cells or animals (Cao et al., 2015; Hoang-Minh et al., 2018; Razzauti and Laurent, 2021; Wang J. et al., 2021), or in the control of ciliary length and composition, notably through the discard of ciliary components (Long et al., 2016; Nager et al., 2017; Phua et al., 2017; Salinas et al., 2017; Akella et al., 2020; Razzauti and Laurent, 2021) (see below).

### Intercellular communication via the release of ciliary EVs

Once released into the extracellular environment, ciliary EVs exert remote effects on recipient cells, as described in lower species such as *Trypanosoma brucei*, *Chlamydomonas* or *Caenorhabditis elegans* (Wood et al., 2013; Wang et al., 2014; Schorey et al., 2015; Szempruch et al., 2016a; Wang and Barr, 2018; Carter and Blacque, 2019; Luxmi and King, 2022). *Trypanosoma brucei rhodesiense* produces EVs containing serum resistance-associated protein (SRA), which confers resistance to human innate immunity, by neutralizing the circulating trypanosome lytic factors (TLF). Co-culture experiments have shown that EVs deriving from flagella in *T.b. rhodesiense* transfer SRA to *T.b. brucei* and confer resistance to TLF. For *Chlamydomonas*, flagella are an important source of EVs that are involved in different stages of their life cycle. Indeed, during the vegetative phase, the release of EVs containing an active enzyme (vegetative lytic enzyme protease) allows the digestion of the extracellular matrix (cell wall) of the sporangial cell and the release (“hatching”) of daughter cells (Wood et al., 2013). The authors confirmed the activity of the enzyme by exposing ciliary EVs isolated from wild type *Chlamydomonas* to sporangia from aflagellar IFT88 null mutants. While mutants were unable to hatch, ciliary EVs restored this function (Wood et al., 2013). Among the bioactive molecules transported by *Chlamydomonas* ciliary EVs, the membrane polypeptide SAG1-C65 may contribute to this mating success (Cao et al., 2015). Such a role of ciliary EVs in the control of reproductive functions is also observed in *C. elegans*, where ciliated sensory neurons release polycystin-containing EVs that affects mating related locomotory behaviors in males (Wang et al., 2014; Wang and Barr, 2018). As a consequence of mating, the male will also modulate the composition of EVs released into its surrounding environment, with an increase in the ratio of PKD-2 to CIL-7 EVs depending on the type of populations mated (isolated virgin males vs. mixed males) (Wang J. et al., 2021). In the cephalic sensory organ of *C. elegans*, ciliated neurons release EVs from PCMC that are phagocytosed by adjacent glial cells. This contributes to the maintenance of the biochemical integrity of the cilium. This intercellular communication mediated by ciliary EVs has been proposed to participate to the morphogenesis of this sensory organ (Akella et al., 2020; Razzauti and Laurent, 2021). Yet, in mammals, such a role for ciliary EVs in intercellular communication remains elusive. A homeostasis function for ciliary EVs has been suggested in the cardiovascular system, where a decrease in the number of circulating ciliary EVs is associated with abnormalities in blood pressure and heart rate (Mohieldin et al., 2020). In bbs4^−/−^ and bbs8^−/−^ mutants, where intraflagellar transport is interrupted, some of the proteins originally destined to be released through large ciliary EVs were found enriched in small EVs deriving from the cell cytoplasm, suggesting the existence of a tight connection between EVs released from different cell compartments (Volz et al., 2021). These protein- and miRNA-enriched EVs were able to modulate Wnt signaling in recipient cells (Volz et al., 2021), confirming their role in intercellular communication. Co-incubation of culture medium containing primary ciliary EVs from cancer cells with glioma cells increased their proliferation. This was confirmed by comparison with culture media from cells lacking primary cilia such as KIF3A^−/−^ or IFT88^−/−^ (Hoang-Minh et al., 2018).

Finally, while the role of the primary cilium as a signaling antenna is well known (Satir et al., 2010; Pala et al., 2017; Wheway et al., 2018; Anvarian et al., 2019), it may also be able to receive signals in the form of EVs or vesicle-like particles. While several microscopy studies have shown an association between EVs and primary cilia (Table 1B), *in vitro* incubation of isolated urinary PKD positives EVs with primary cilia from sections of rat biliary tree and IMCD3 cells confirmed this interaction, suggesting that the EV-ciliary interaction might also support intercellular communication (Hogan et al., 2009). For instance, EVs isolated from rat biliary tree and incubated with ciliated cholangiocytes decreased their proliferation through the disruption of the ERK signaling pathway and miR-15 A expression (Masyuk et al., 2010).

### Control of ciliary length and composition

In BBS8 and osm-3 (anterograde motor kinesin 3) *C. elegans* mutants, the release of ciliary EV is increased at both the tip of the cilium and the PCMC (Akella et al., 2020; Razzauti and Laurent, 2021), where they serve as a safety valve to maintain the cilium composition. Similarly, when intraflagellar trafficking is blocked in mammalian cells, the number of activated GPCR-like receptors is regulated through their removal by ectocytosis, a mechanism that modulates cell signaling responses (Nager et al., 2017). In addition, by releasing EVs, the cell promotes cilium disassembly and re-entry into mitosis, which is confirmed by direct blockade of actin polymerization or knockdown of actin regulators (Phua et al., 2017; Wang et al., 2019). This disassembly of the cilium is especially important because targeted removal of intraflagellar transport components (*e.g.,* ARL13B, IFT proteins, ICK) could concomitantly limit primary cilium elongation (Phua et al., 2017; Nakamura et al., 2020). More broadly, the control of cilium length could play an important role in the function of the cilium itself. For example, rod photoreceptors are sensory neurons whose length and function are highly dependent on their cilia. In 1969, Young and Bok showed that the discs at the end of the rod photoreceptors were continuously detached and phagocytosed by cells of the retinal pigment epithelium, thus ensuring the continuous renewal of the photoreceptor outer segment (Young and Bok, 1969). In contrast, the capacity for ectocytosis by the photoreceptor cilium is itself blocked during the cilium formation phase. Indeed, in absence of Peripherin-2 (RDS) in murine rds−/− mutants, the rod exhibits a higher capacity to release EVs, which prevents the formation of discs at the base of the outer segment (Salinas et al., 2017). Thus, the control of ciliary EVs release finely regulates the outer segment organization and turnover.

## Mechanism of release of ciliary EVs

**FIGURE 1:**
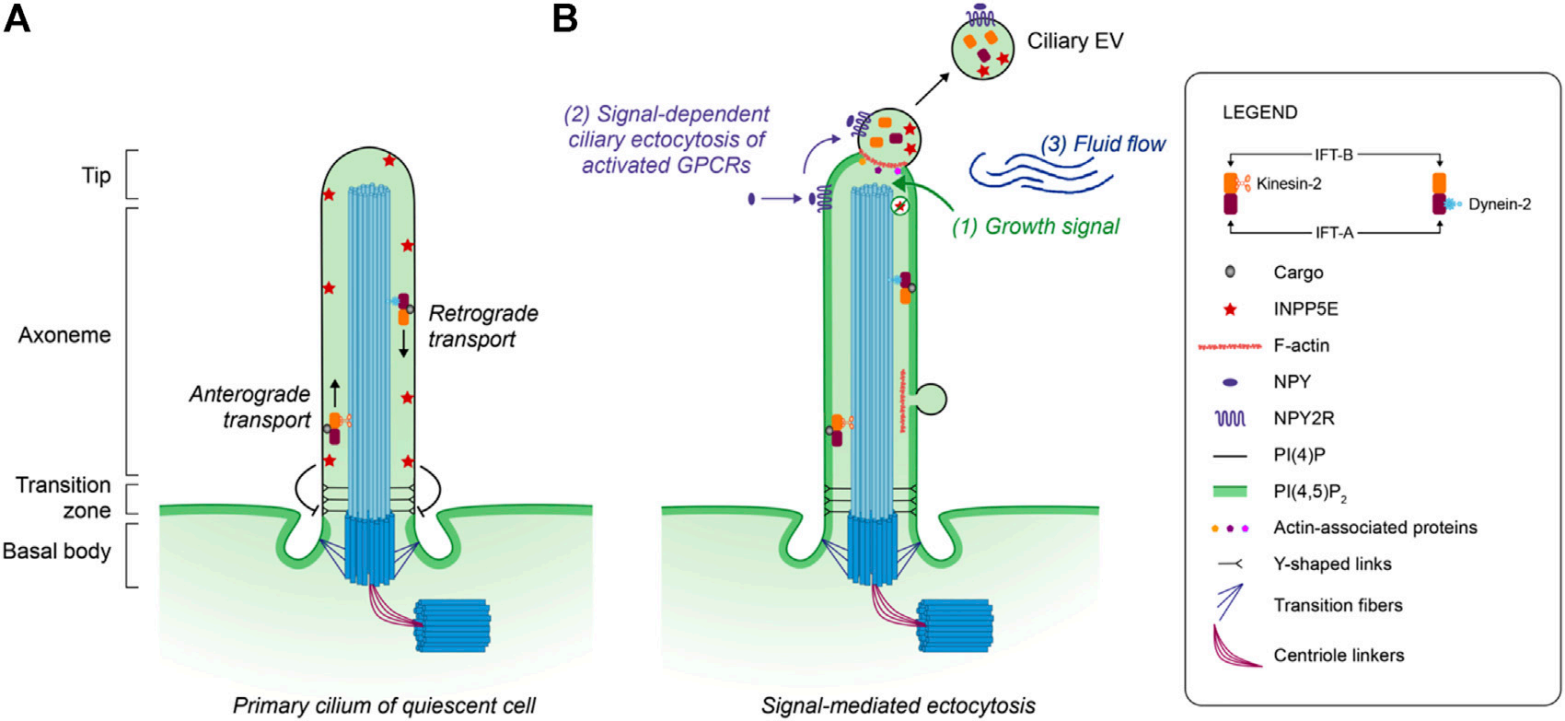
Model of ectocytosis from the mammal primary cilium. **(A)** The intraflagellar transport system (IFT) participates in the assembly and maintenance of the primary cilium through the transport of molecular cargos by IFT-A and IFT-B protein complexes. In quiescent cells, the primary ciliary membrane is enriched in PI(4)P due to the presence of the ciliary phosphatase INPP5E which converts PI(4,5)P2 to PI(4)P. **(B)** Following a proliferation growth signal (1), the ciliary phosphatase INPP5E is removed from the membrane, promoting the redistribution of the phosphoinositide PI(4,5)P_2_ in the primary cilium. These membrane changes allow the local polymerization of F-actin through the recruitment of actin-associated proteins, followed by the ectocytosis of ciliary EVs enriched in bioactive molecules. Although ectocytosis following actin polymerization was identified at the cilium tip, F-actin was also observed in the vicinity of ciliary EVs present along the axoneme membrane. The activated Neuropeptide Y receptor type 2 (NPY2R) uses ectocytosis as a constitutive way to exit the cilium (2). The mechanical force (3) generated by the surrounding fluid flow also promotes the release of ciliary EVs.

While the release of EVs has been observed from different membrane protrusions and cell types, the underlying mechanism of liberation remains largely unknown. The primary cilium, with its unique structural core and composition, is the best described source of protrusion-derived EVs. Primary cilia are assembled via IFT that traffics microtubule clusters and protein cargo along the axoneme. These structures are essential to ciliary genesis and functions and have long overshadowed the possible functions of actin within the primary cilium (Garcia et al., 2018; Brücker et al., 2020; Smith et al., 2020). In fact, microscopy studies have revealed the presence of F-actin along the axoneme and at the ciliary tip (Brücker et al., 2020; Smith et al., 2020) where it controls the release of EVs (Nager et al., 2017; Phua et al., 2017). For instance, live fluorescence imaging on mammalian cells showed that “decapitation” of the primary cilium tip was preceded by a transient actin polymerization at the site of excision (Phua et al., 2017). In addition, the use of actin poisons prevented the formation of EVs enriched in GPCRs such as neuropeptide Y receptor 2 (NPY2R), supporting a role for actin in the ciliary EV release process (Nager et al., 2017). Recently, a cryo-electron tomography (cryo-ET) study also revealed the presence of EVs in the vicinity of primary cilia isolated from MDCK-II cells. Among the EVs detected, 50% colocalized with actin filaments (Kiesel et al., 2020). The role of actin polymerization is further supported by the recruitment of actin-associated proteins such as Myosin 6, Drebrin, Cofilin-1, Rab7 to the site of EV release (Nager et al., 2017; Phua et al., 2017; Wang et al., 2019). Proteomic analyses confirmed the presence of many actin-related proteins in both the primary cilium and ciliary EVs (Ishikawa et al., 2012; Mick et al., 2015; Kohli et al., 2017; Mohieldin et al., 2021b; Volz et al., 2021), and the detection of these proteins is decreased in urinary EVs of nephronophthisis-related ciliopathy patients (Stokman et al., 2019). However, the functional link between actin-regulators and actin depolymerization inside the cilium requires further exploration. The local and transient actin polymerization occurring at the tip of primary cilia is thought to be coupled with a change in the phosphoinositide (PI-P) composition of the ciliary membrane. The primary cilium membrane of a quiescent cell is enriched in PI(4)P due to the presence of the ciliary phosphatase INPP5E (Inositol Polyphosphate-5-Phosphatase E) (Figure 1A), which converts PI(4,5)P_2_ to PI(4)P (Chávez et al., 2015; Garcia-Gonzalo et al., 2015). Following ciliary depletion of INPP5E after a proliferative signal, redistribution of PI(4,5)P_2_ into the cilium is observed (Phua et al., 2017). PI(4,5)P_2_-dependent actin regulators, including Cofilin-1, Fascin, Kras and SNX9 regulate actin polymerization and subsequent ciliary EV release and deciliation (Phua et al., 2017, 2018). Many molecular factors involved in microtubule stabilization and destabilization (Pugacheva et al., 2007; Plotnikova et al., 2015) can link growth signals, membrane events, and the evolution of actin and microtubule cytoskeletons. While this model provides an outline of the process leading to the release of ciliary EVs, other factors such as the mechanical fluid shear stress appears to be sufficient to generate ciliary EVs in 85% of cilia (Figure 1B) (Mohieldin et al., 2020). This process, confirmed by several simultaneous approaches (*e.g.,* microscopy, proteomics), has been reproduced in endothelial cells (Mohieldin et al., 2021b). As primary cilia are sensory antennae exposed to bodily fluids and their turbulence, the implications of this shear force deserve special attention in future studies. Furthermore, recent research on mutants of proteins involved in IFT raises the possible implications of this transport in the release of ciliary EVs. In normal conditions, the combined action of BBSome, the small GTPase ARL6 and the activated GPCR sensor β-arrestin 2, sequesters the ciliary somatostatin receptor 3 (SSTR3) into the cell (Nager et al., 2017; Ye et al., 2018; Nachury and Mick, 2019). In knockdown or knockout models for BBSome, ARL6 or IFT-B component, retrograde IFT functions are impaired, and SSTR3 accumulates at the ciliary tip before undergoing ectocytosis. These studies therefore support the link existing between BBSome and IFT trains in the control of EV release (Nager et al., 2017; Wang W. et al., 2021; Jordan and Pigino, 2021). EV composition could provide information about the possible involvement of the IFT system in the genesis of ciliary EVs. Indeed, IFT-B is more abundant than IFT-A in EVs released from the primary cilium (Phua et al., 2017; Nakamura et al., 2020), and a defect in BBsome-mediated protein trafficking leads to a transfer of a portion of large EV-specific proteins to small EVs (Volz et al., 2021). Although the mutations of genes that encode IFT proteins lead to a wide variety of ciliopathies (Pigino, 2021), the direct relationship between these diseases and abnormal production of ciliary EVs has never been explored.

## Ciliary EV markers

**FIGURE 2:**
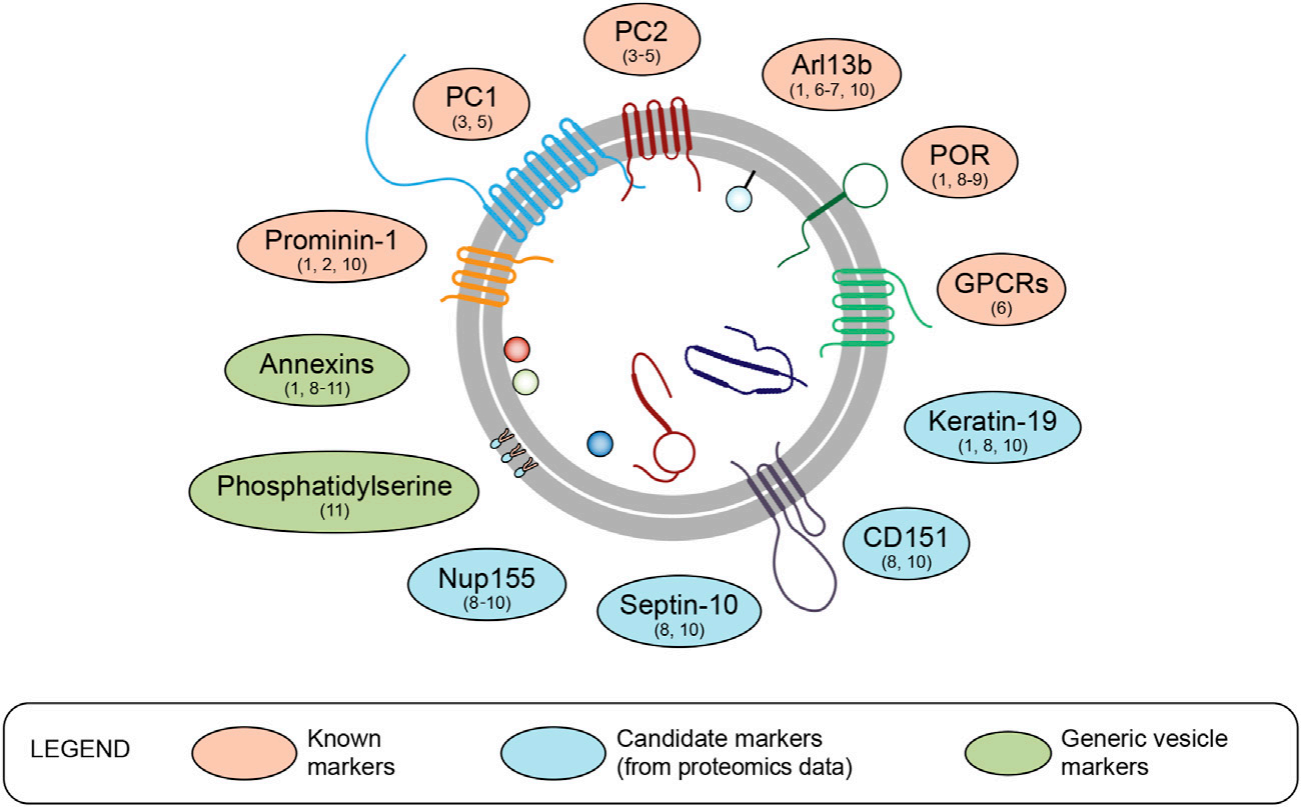
Markers and candidate markers of ciliary extracellular vesicles. With the increasing interest in ciliary EVs, the identification of specific markers is needed. Although ciliary EVs are mostly large EVs (ectosomes), classical markers of large EVs are not found in ciliary EVs, except for annexins and phosphatidylserine. The cross comparison of proteomic data from the primary cilium and ciliary EVs allowed the identification of several potential markers, in addition to those already described in the literature. References: ^1^ Ishikawa et al., (2012), ^2^ Dubreuil et al., (2007), ^3^ Yoder et al., (2002), ^4^ Pazour et al., (2002), ^5^ Hogan et al., (2009), ^6^ Nager et al., (2017), ^7^ Phua et al., (2017), ^8^ Mohieldin et al., (2021b), ^9^ Volz et al., (2021), ^10^ Mick et al., (2015), ^11^ Théry et al., (2018)

With the growing study of EVs in all bodily fluids, structural and biochemical characterization is needed to distinguish true vesicles from artefactual protein aggregates or cellular debris. For that purpose, consensual guidelines that define the optimal methodologies and relevant markers have been established for the study of EVs, based on the features of cytosolic-derived EVs from human and mouse species (Théry et al., 2018). While the definition of an unambiguous nomenclature to categorize EVs has been attempted, a lack of consensus on the definition of EV markers persists (Théry et al., 2018). This is in part due to the fact that the molecular composition of vesicles varies depending on the model organism/cell type studied, its patho-physiological state and the source/mode of release from the cell (Haraszti et al., 2016; Tricarico et al., 2016; Martínez and Andriantsitohaina, 2017). Extracellular vesicles deriving from cell protrusions, including primary cilia, appear as a new type of EVs that are associated with specific ciliary markers (Figure 2).

While the number of studies performed on ciliary EVs is still limited, approaches have been developed to specifically identify ciliary markers. For instance, the comparative proteomic study of EVs isolated from ciliated wild-type (WT) and non-ciliated *IFT88* knockout (KO) mouse endothelial cells underscored the different composition of ciliary and cytoplasmic EVs and identified specific markers of ciliary EVs (Mohieldin et al., 2021b). In this study, prevailing large EV markers such as actinin-1 and 4, MVP, and eEF2 (Mathivanan et al., 2010; Kowal et al., 2016; Théry et al., 2018) were not found in ciliary EVs (Mohieldin et al., 2021b). However, NADPH cytochrome P450 reductase (POR) was detected at the tips of primary cilia and enriched in ciliary EVs (Mohieldin et al., 2021b). POR participates in the peroxidation of membrane phospholipids, resulting in plasma membrane rupture in the context of cell death (Zou et al., 2020), but additional studies are needed to define the contribution of POR at the site of EV release within the primary cilium. Furthermore, the extent to which POR can be used as a universal marker of ciliary EVs remains to be established. In addition to POR, Prominin 1 (PROM1, also known as CD133) is a protein associated with different types of protrusions (*e.g.,* filopodia, microvilli, primary cilium, and photoreceptor-specific outer segments) (Jászai et al., 2020) that is also found enriched in EVs derived from primary cilia and microvilli (Marzesco et al., 2005; Dubreuil et al., 2007). Similarly, Polycystin 1 (PC1) and Polycystin 2 (PC2) are two proteins that form a calcium-permeable ion channel in the membrane of the primary cilium, and are associated with a subpopulation of EVs found in urine (Pazour et al., 2002; Yoder et al., 2002; Hogan et al., 2009). The ADP ribosylation factor-like GTPase ARL13b (Nachury and Mick, 2019), a well-known marker of the primary cilium (Nager et al., 2017; Phua et al., 2017) and photoreceptors (Dilan et al., 2019), is used to label ciliary EVs (Table 1). Other compounds present at the ciliary membrane (*e.g.,* GPCRs, INPP5E) are found concentrated in ciliary EVs following their release from the cilium tip (Nager et al., 2017; Phua et al., 2017; Nachury and Mick, 2019). Cross comparison of proteomics data performed on primary cilia organelles with those of ciliary EVs in mammals generated a list of potential markers (Ishikawa et al., 2012; Mick et al., 2015; Mohieldin et al., 2021; Volz et al., 2021), including Keratin-19, Nup155, Septin-10 and CD151. Among families of proteins and phospholipids (*e.g*., annexins and phosphatidylserine) that are used as EV markers (Rousseau et al., 2015; Théry et al., 2018; Rogers et al., 2020), annexin A8 has also been identified in ciliary EVs (Mohieldin et al., 2021b).

Apart from their protein composition, large and small EVs deriving from the cell cytosolic compartment are also enriched in other bioactive molecules, including small non-coding RNAs (sncRNAs) and lipids (Choi et al., 2013; Meldolesi, 2018; Volz et al., 2021) that participate in intercellular communication. While limited information is available regarding the molecular cargo transported by ciliary EVs and its fate, Volz et al. (2021) found an impaired sorting of bioactive molecules between subpopulations of EVs following ciliary dysfunction, pointing to the role of ciliary EVs in the modulation of cellular signaling.

## Conclusion

The expanding field of research on EVs has entailed their characterization, the definition of a consensual nomenclature (Théry et al., 2018), and their functional analyses in distinct model systems and organisms (Yáñez-Mó et al., 2015). As carriers of the patho-physiological molecular signature of their cell of origin, EVs detected in all bodily fluids constitute attractive targets for non-invasive diagnosis of human pathologies (Kosaka et al., 2019). The growing interest in EVs derived from cellular protrusions, particularly ciliary EVs, opens the door to new fundamental questions: to what extent do ciliary EVs share common protein and lipid markers, cargo packaging mechanisms, features, and functions with cytosol derived EVs? Do they participate in the control of recipient cell functions through cellular crosstalk, as described in invertebrates and unicellular organisms? Or do they assist exclusively in the removal of bioactive compounds from the donor cell to control its function? Cilia are ubiquitous organelles important to the homeostatic control of biological systems, and their dysfunction causes ciliopathies. In addition, the primary cilium acts as a cellular brake upon cell division, and growing evidence points to its role in cancer initiation and progression (Izawa et al., 2015; Gerhardt et al., 2016; Eguether and Hahne, 2018). With these considerations in mind, additional investigations are needed to determine if the composition and number of ciliary EVs detected in bodily fluids can indicate the patho-physiological status of the cells, and if ciliary EVs could serve as new targets for the non-invasive diagnosis of cilia-related pathologies.
